# Electrodermal Activity Is Sensitive to Sleep Deprivation but Does Not Moderate the Effect of Total Sleep Deprivation on Affect

**DOI:** 10.3389/fnbeh.2022.885302

**Published:** 2022-07-04

**Authors:** Courtney A. Kurinec, Anthony R. Stenson, John M. Hinson, Paul Whitney, Hans P. A. Van Dongen

**Affiliations:** ^1^Department of Psychology, Washington State University, Pullman, WA, United States; ^2^Sleep and Performance Research Center, Washington State University, Spokane, WA, United States; ^3^Elson S. Floyd College of Medicine, Washington State University, Spokane, WA, United States

**Keywords:** electrodermal lability, physiological arousal, affect, vigilant attention, sleepiness, mood, experienced emotion

## Abstract

Emotion is characterized by dimensions of affective valence and arousal, either or both of which may be altered by sleep loss, thereby contributing to impaired regulatory functioning. Controlled laboratory studies of total sleep deprivation (TSD) generally show alterations in physiological arousal and affective state, but the relationship of affect and emotion with physiological arousal during TSD has not been well characterized. Established methods for examining physiological arousal include electrodermal activity (EDA) measures such as non-specific skin conductance responses (NSSCR) and skin conductance level (SCL). These measures are robust physiological markers of sympathetic arousal and have been linked to changes in experienced emotion. To explore the link between physiological arousal and affect during sleep deprivation, we investigated individuals’ EDA under TSD and its relationship to self-reported affect. We also investigated the relationship of EDA to two other measures known to be particularly sensitive to the arousal-decreasing effects of TSD, i.e., self-reported sleepiness and performance on a vigilant attention task. Data were drawn from three previously published laboratory experiments where participants were randomly assigned to either well-rested control (WRC) or 38 h of TSD. In this data set, comprising one of the largest samples ever used in an investigation of TSD and EDA (*N* = 193 with 74 WRC and 119 TSD), we found the expected impairing effects of TSD on self-reported affect and sleepiness and on vigilant attention. Furthermore, we found that NSSCR, but not SCL, were sensitive to TSD, with significant systematic inter-individual differences. Across individuals, the change in frequency of NSSCR during TSD was not predictive of the effect of TSD on affect, sleepiness, or vigilant attention, nor was it related to these outcomes during the rested baseline. Our findings indicate that while physiological arousal, as measured by EDA, may be useful for assessing TSD-related changes in non-specific arousal at the group level, it is not associated with individuals’ self-reported affect at rest nor their change in affect during TSD. This suggests that an essential aspect of the relationship between physiological arousal and self-reported affect is not well captured by EDA as measured by NSSCR.

## Introduction

Multiple sources of evidence demonstrate that insufficient sleep alters the experience of emotion. Sleep loss has been found to decrease positive mood (Pilcher and Huffcutt, 1996; Dinges et al., 1997) and increase negative mood states (Scott et al., 2006; Kahn-Greene et al., 2007), and has been linked to difficulties in regulating emotion (Lustig et al., 2021; Lustig, 2021; Stenson et al., 2021). The experience of emotion is characterized by affective valence (positive/negative) and by the level of arousal (how intensely an affective state is experienced; Russell, 1980; Posner et al., 2005). Even though arousal is an essential component of emotion, studies of how emotional experience is influenced by sleep loss have generally been limited to self-report measures of arousal, with changes in arousal examined separately from emotion. As subjective measures are subject to cognitive biases (Podsakoff et al., 2003), to better understand the impact of sleep deprivation on emotion, there is a need to measure changes in emotion-related arousal due to sleep loss in a more direct and objective way. Such an objective measure of arousal during sleep loss would also be useful for monitoring personnel alertness in fields where individuals are remotely located and experience extended wake periods.

It is well-established that sleep deprivation decreases arousal, as the build-up of homeostatic sleep drive with time awake increases the pressure for sleep (modulated by circadian rhythm; Borbély et al., 2016). Individuals show increased sleepiness and fatigue on subjective and objective measures when sleep deprived (Daan et al., 1984; Oken et al., 2006; Åkerstedt et al., 2014) but across individuals, the increases in subjective sleepiness are distinct from those in objective measures (Van Dongen et al., 2004a; Franzen et al., 2008). Despite purportedly assessing the same construct, subjective and objective measures related to arousal often show a negative correlation (Danker-Hopfe et al., 2001) or no reliable association (Seidel et al., 1984)—a dissociation that extends to conditions of sleep deprivation (Leproult et al., 2003; Van Dongen et al., 2004a). A reasonable conclusion from these results is that subjective measures of arousal are unlikely to serve as adequate substitutes for more direct measures of physiological arousal and are correspondingly inadequate to assist in explaining objective changes in the experience of emotion due to sleep deprivation.

An objective measure that may be more useful for assessing an individual’s physiological arousal and changes in mood during sleep deprivation is electrodermal activity (EDA). EDA provides a well-established method for assessing physiological arousal by measuring changes in current passed through two points of contact across the skin (Dawson et al., 2017). Unlike other measures of physiological arousal (e.g., EEG, heart rate, pupillary response) which are subject to a variety of influences by both sympathetic and parasympathetic activity, the activity of the sweat glands that determine electrical conductance across the skin is directly driven by the sympathetic nervous system. Thus, EDA provides a relatively unadulterated measure of sympathetic arousal. As sleep deprivation is thought to increase sympathetic tone and decrease parasympathetic tone (McEwen, 2006), using EDA as opposed to other measures of physiological arousal avoids the potential that these two nervous systems will exert confounding influences.

There are several indices drawn from EDA data with different functional properties. For changes in arousal in response to continuous situations such as sleep deprivation, the most useful electrodermal measures are skin conductance level (SCL) and non-specific skin conductance responses (NSSCR). SCL represents the tonic level of skin conductance, and NSSCR are skin conductance responses that occur without a specific eliciting stimulus (Dawson et al., 2017). Increases in either SCL or the frequency of NSSCR reflect increases in sympathetic activation, usually due to changes in task situation (e.g., anticipating or beginning performance). Notably, research has found that these measures of EDA may also be linked to experienced emotion, as individuals show increased SCL and NSSCR during emotion regulation (Gross and Levenson, 1993; Gross, 1998; Egloff et al., 2006; Duijndam et al., 2020) and following procedures to induce emotion over extended periods (Kreibig et al., 2007). More importantly, SCL and frequency of NSSCR have been found to be associated with changes in subjective arousal in rested individuals rating their emotional reactions to emotional pictures or films, such that SCL and NSSCR increase as emotional arousal ratings increase (Gomez et al., 2016; Duijndam et al., 2020; Rattel et al., 2020; Sato et al., 2020). Even without emotional stimuli, individuals report increased ratings of negative emotion during periods preceded by NSSCR compared to control periods with no preceding NSSCR, suggesting that NSSCR may be linked to more transient experiences of emotion (Nikula, 1991). Additionally, SCL and frequency of NSSCR are decreased in individuals with depression, which is characterized by long-term decreased mood (Iacono et al., 1984; Schwerdtfeger and Rosenkaimer, 2011). Therefore, these measures of EDA may be well-suited for assessing changes in physiological arousal as a component of experienced affect during sleep deprivation.

In addition to capturing physiological responses during exposure to more long-term stimuli or situations (as opposed to discrete stimuli or situations), SCL and NSSCR are also believed to exhibit systematic inter-individual differences (Crider, 1993; Crider et al., 2004; Vaidyanathan et al., 2014). Individual measures of tonic EDA vary widely; according to Dawson et al. (2017), individual SCL typically ranges from 2 to 20 microsiemens (μS), and individual frequency of NSSCR ranges from 1 to 3 per min. Further, these tonic measures are influenced by factors such as age, with older adults showing lower values than younger adults (Surwillo and Quilter, 1965; Barontini et al., 1997); sex, with females generally showing higher levels than males (Kopacz and Smith, 1971; Ketterer and Smith, 1977) and female tonic EDA differing by stage of the menstrual cycle (Gómez-Amor et al., 1990; Goldstein et al., 2005); and ethnicity, with Black Americans showing lower tonic EDA than non-Black Americans (Juniper and Dykman, 1967; Korol et al., 1975; Kredlow et al., 2017). Based on the frequency with which they show NSSCR (or alternatively, based on how quickly individuals habituate and show decreased skin conductance responses to a given stimulus), individuals can be classified as either electrodermally labile or stable. Individuals who are labile show more NSSCR (or slow habituation), whereas stable individuals show fewer NSSCR (or fast habituation; Dawson et al., 2017). Individuals also sometimes show differences in emotional reactivity based on their electrodermal lability classification, such that labiles experience greater affective reactions than stables (Choi et al., 2012, 2015). Electrodermal lability measured by NSSCR or habituation is thought to reflect a single latent phenotype that is influenced by specific genetic and environmental factors (Crider et al., 2004). Electrodermal lability measures have been found to show high test-retest reliability (NSSCR *r* = 0.76, Schell et al., 2002; NSSCR *r* = 0.70, Crider et al., 2004) and are stable across time points (NSSCR ICC = 0.77, Bari, 2019). Research on twins has found that around 50% of the variance in electrodermal lability is heritable (Isen et al., 2012; Vaidyanathan et al., 2014). If there are systematic interindividual differences in electrodermal lability or tonic EDA more generally during sleep deprivation, it would suggest that some individuals are more vulnerable to sleep deprivation-related changes in EDA than others, and as such, should also show corresponding changes in self-reported affect. Further, the presence of systematic inter-individual differences would make the findings at the group level less reliable, as they could mask these individual-level effects.

Interest in whether electrodermal lability reflects systematic interindividual differences arose from work demonstrating that individuals differ by lability classification on certain cognitive tasks. For instance, individuals classified as electrodermally labile have been found to show better performance on vigilant attention tasks (Crider and Adgenbraun, 1975; Sostek, 1978; Munro et al., 1987) and simple response time (RT) tasks (Vossel, 1988; Wilson and Graham, 1989) than individuals classified as electrodermally stable. These same vigilant attention and simple RT tasks are also closely associated with changes in arousal during sleep deprivation (Ratcliff and Van Dongen, 2011), showing large sleep deprivation effects (Lim and Dinges, 2010) with substantial interindividual differences (Van Dongen et al., 2004a). Thus, in addition to affecting experienced emotion, sleep deprivation effects on EDA and physiological arousal more broadly may also affect other measures closely linked to changes in arousal, like vigilant attention.

The relationship between electrodermal lability or EDA measures and sleep deprivation has been previously investigated, but older studies used a variety of methodologies and sometimes employed extremely small sample sizes, yielding mixed findings (see Horne, 1978). However, recent work has produced more reliable results. For example, studies found that, as time awake increases, skin resistance level (the inverse of SCL) increases (Miró et al., 2002) and the frequency of NSSCR decreases (Posada-Quintero et al., 2017, 2018), although the change in NSSCR may be limited to electrodermal labiles (Michael et al., 2012). At least one study examined the relationship between EDA during sleep deprivation and emotion (Liu et al., 2015), but the inclusion of a crossed stressor condition in the absence of a no-stress control makes it difficult to determine whether changes in physiological arousal during sleep deprivation alone influenced experienced emotional states. Other studies showed that measures of EDA are associated with self-reported arousal and cognitive performance during sleep loss. Skin resistance level was observed to be positively correlated with both subjective sleepiness and performance on a simple RT task (Miró et al., 2002), and NSSCR were found to be negatively correlated with lapses on the psychomotor vigilance test (PVT; Dinges and Powell, 1985), the gold standard for assessing behavioral alertness during sleep deprivation (Posada-Quintero et al., 2018). These relationships may be moderated by lability, as electrodermal labiles have been found to show higher subjective sleepiness during total sleep deprivation than stables (Michael et al., 2012). These more recent studies still rely on relatively small samples (ranging from 10 to 40), but more critically for the present purposes, they do not make any direct connection between changes in electrodermal lability or EDA under sleep deprivation and changes in mood or affective state.

To clarify the relationship between physiological arousal and emotional states during sleep deprivation, we gathered data from three in-laboratory sleep deprivation protocols on which we have previously published (e.g., Whitney et al., 2017; Honn et al., 2019; Lawrence-Sidebottom et al., 2020; Kurinec et al., 2021; Stenson et al., 2021). With these data, which formed one of the largest samples used in an investigation of total sleep deprivation (TSD) and EDA, we investigated the relationship between EDA, as measured by NSSCR frequency and SCL, to experienced emotion, operationalized as self-reported affect, under TSD. Specifically, we investigated how TSD influences NSSCR and SCL, whether EDA shows systematic interindividual differences during sleep deprivation, and whether a change in EDA due to TSD is related to change in affect. Based on previous research, we expected that TSD would lead to decreases in tonic EDA and that vulnerability to these TSD-related changes would be a stable interindividual difference. Additionally, because less labile individuals have been found to show less emotional reactivity, we expected that the size of the decrease in affect during sleep deprivation would be positively associated with the level of decrease in EDA during TSD. Secondarily, we explored how changes in NSSCR frequency and SCL are associated with changes in other measures closely related to arousal, namely self-reported arousal, and vigilant attention, during TSD. Given that interindividual differences in lability have been linked to interindividual differences in sleepiness and vigilant attention, we anticipated that the level of decrease in these measures during TSD would be positively associated with the size of the observed decrease in EDA.

## Materials and Methods

### Participants

Participants (*N* = 193, 51.8% female, 83.9% White, 93.3% right-handed) were drawn from three in-laboratory protocols (*N*_1_ = 62, 53.2% female, 90.3% White, 95.2% right-handed; *N*_2_ = 54, 46.3% female, 87.0% White, 96.3% right-handed; *N*_3_ = 77, 54.5% female, 76.6% White, 89.6% right-handed). These protocols were selected as they all included EDA measurements, contained relatively large samples, and used the same duration of TSD. Participants’ ages ranged from 21 to 40 years (*M* = 26.8, *SD* = 4.8). All participants were screened to be physically and psychologically healthy and were free of drugs (except oral contraceptives); were not currently receiving medical treatment or pregnant; did not have any sleep or circadian disorders; had not traveled across time zones within 1 month or engaged in shift work within 3 months; and had normal or corrected to normal vision and hearing. During the week prior and during the study, participants refrained from caffeine, tobacco, drug, and alcohol use, as verified by a urine screening and breathalyzer. Participants were asked to maintain their habitual sleep schedule during the week prior to the study and to refrain from napping. Adherence to sleep schedule was verified by sleep diary, called-in sleep and wake times, and wrist-worn actigraphy.

Participants were randomly assigned to either a total sleep deprivation (TSD; *n* = 119) or a well-rested control (WRC; *n* = 74) condition. In the first protocol, the probability to be assigned to either condition was 0.50 (34 TSD, 28 WRC). In the latter two protocols, the probability to be assigned to the TSD condition was set to 0.67 as part of other investigations unrelated to the present study (Protocol 2: 36 TSD, 18 WRC; Protocol 3: 49 TSD, 28 WRC).

The procedures involved in the three protocols were approved by the Washington State University Institutional Review Board. Participants gave written informed consent to the procedures before beginning the studies, and they were compensated for their time.

### Procedure

The in-laboratory protocols took place under controlled conditions in the Sleep and Performance Research Center at Washington State University Health Sciences Spokane. Ambient temperature (21 ± 1°C) and light levels during scheduled wakefulness (<100 lux) were fixed. For all three protocols, participants were in the laboratory for 4 days (three nights). Participants entered the laboratory in the late afternoon on day 1, and all participants had a 10-h (22:00–08:00) baseline sleep opportunity. On the evening of day 2, participants were informed of their condition assignments. Those in the TSD condition were kept awake for 38 h, whereas those in the WRC condition had another 10-h sleep opportunity. On day 3, all participants had a 10-h (recovery) sleep opportunity before leaving the laboratory on day 4. Up to four individuals participated at the same time, and participants were assigned to separate rooms for sleep and performance testing. Meals were provided every 4 h during scheduled wakefulness. When participants were not scheduled to sleep or not performing testing or having meals, they were allowed to engage in non-vigorous activities, such as watching innocuous movies or reading. Vigorous activities, such as exercise, were prohibited. Laptops, tablets, cell phones, live television, live radio, or other means of interacting outside the laboratory environment were not permitted, and visitors were not allowed. Participants’ behavior was monitored continuously by trained research assistants to ensure compliance.

EDA data were collected from each protocol only before the start of morning and afternoon cognitive testing sessions on days 2 and 3. In the first protocol, morning testing started at 10:00, and afternoon testing started at 14:00. In the second protocol, morning testing started at 09:30, and afternoon testing started at 15:00. Finally, in the third protocol, morning testing started at 09:45, and afternoon testing started at 14:30 on day 2 and at 14:00 on day 3. Because of differences in the task batteries specific to each protocol, the protocols combined in this study started SCL measurement at slightly different times, which is the reason for the modest variability in SCL start times.

Self-reported affect, self-reported sleepiness, and vigilant attention were assessed every 2–4 h during scheduled wakefulness. Only morning and afternoon test bouts closest to the EDA recordings and shared across all three protocols, at 09:00 and 13:00 on days 2 and 3, were included in analyses to minimize circadian confounds in comparisons with EDA data.

### Materials

#### Electrodermal Activity (EDA)

EDA measures of interest were mean SCL and NSSCR frequency. EDA was based on continuous sampling of SCL at 50 Hz during a 5-min interval at rest, when participants were not engaged in any tasks, prior to the beginning of the aforementioned morning and afternoon cognitive test sessions on days 2 and 3. EDA measurements from Protocols 1 and 2 were recorded with a Psychlab SC5 24-bit system (Contact Precision Instruments, Cambridge, MA), and data from Protocol 3 were recorded with a BIOPAC recording system (BIOPAC Systems, Inc., Goleta, CA). Disposable self-adhesive electrodes filled with isotonic gel for consistent ohmic contact were attached to the anterior surface of the non-dominant hand on the intermediate phalange of the index and middle fingers. Electrodes, which were the same size across protocols, were attached to the recording system through leads with pinch connectors, and real-time SCL was observed to ensure the electrodes were connected correctly and that SCL greater than zero was produced. Once the electrodes were attached, participants were instructed to keep their hands still, relax, and to avoid excessive movement while preparing for the forthcoming experimental test battery. Each participant’s SCL was plotted and visually inspected before conducting any further analyses.

Mean SCL was computed across the 5-min sampling interval. NSSCR were calculated in each successive 10-s bin of the 5-min sampling interval. SCL at the beginning of each 10-s bin served as a reference measure, which was subtracted from the peak SCL during the remaining 10 s. All SCL values above 0.10 μS were analyzed; more than 99.7% of all samples were included. An obtained SCL amplitude ≥ 0.05 μS above reference was recorded as an NSSCR for that bin. Less than 2% of all SCL samples were below 0.50 μS, although two participants’ SCL were consistently below 0.50 μS. However, as omitting these data points did not materially affect the subsequent analyses, we chose to keep the widest range of SCL values for this study and did not exclude the two participants. Any peak SCL of greater than 3 μS was considered to be an artifact of movement and was excluded. Such events were rare, i.e., <1% of all samples. The frequency of NSSCR was assessed as the number of bins flagged as having an NSSCR, with a maximum possible frequency of 30 across the 5-min sampling interval. We observed the vast majority of NSSCR in the range from 5 to 15 during the 5-min sampling interval, which is consistent with the typically reported range of 1–3 NSSCR per minute (Dawson et al., 2017).

#### Positive and Negative Affect Schedule (PANAS)

The PANAS (Watson et al., 1988) is a self-report questionnaire that assesses an individual’s mood state at the present moment. The PANAS consists of two 10-item subscales designed to measure positive and negative affect. On each subscale, participants indicate the degree to which positive emotions (e.g., inspired) or negative emotions (e.g., distressed) describe their current emotional state using a 5-point Likert-type scale (1 = Very slightly to 5 = Extremely). The PANAS provides total scores for each subscale, ranging from 10 to 50, such that higher scores indicate greater positive or negative affect. Valence and arousal are deliberately intertwined on this instrument (Watson and Tellegen, 1985). The PANAS shows good internal consistency and construct validity (Watson et al., 1988; Crawford and Henry, 2004) and has been previously used to assess changes in self-reported mood during TSD (Franzen et al., 2008; Riedy et al., 2013; Stenson et al., 2021).

#### Karolinska Sleepiness Scale (KSS)

The KSS (Åkerstedt and Gillberg, 1990) is a self-report scale for assessing subjective sleepiness. On this measure, participants indicate their current level of sleepiness using a 9-point Likert-type scale (1 = Very alert; 9 = Very sleepy, great effort to keep awake, fighting sleep). The KSS has high sensitivity to sleep loss (Kaida et al., 2006; Åkerstedt et al., 2014).

#### Psychomotor Vigilance Test (PVT)

The PVT (Dinges and Powell, 1985) is a 10-min serial RT task that assesses an individual’s ability to sustain vigilant attention. On this task, participants respond as quickly as possible (*via* button press) to a visual stimulus that appears on the screen at random intervals of 2–10 s. Vigilant attention was quantified by the log signal-to-noise ratio (LSNR), which is the log-transformed ratio of the power of the relevant information (signal) to the power of the irrelevant information (noise) in the RT distribution (Chavali et al., 2017). Performance on the PVT is highly sensitive to sleep loss (Van Dongen et al., 2003; Lim and Dinges, 2008).

### Statistical Analyses

Individuals were included in analyses if they had available morning or afternoon NSSCR or SCL data for both days. There were 152 participants with both morning and afternoon data, 19 with only morning data, and 22 with only afternoon data; all had SCR data on both measurement days. In these data there were a total of 15 instances distributed over *n* = 9 participants (all from the TSD condition) with NSSCR values of zero (2.25% of all cases); however, these participants also had non-zero SCL for these instances, indicating that the electrodes were recording. Removing these zero NSSCR cases from analyses did not fundamentally change the results; therefore, these participants were retained in the analyses below. Using their sampling data from day 2 (baseline day), participants were then classified separately for the morning and afternoon sampling intervals as electrodermally labile or stable (Morning: labile = 86, stabile = 85; Afternoon: labile = 80, stable = 82) based on median split (Morning Median = 0.31; Afternoon Median = 0.25). A median split was used in accordance with how electrodermal lability has been traditionally defined in the literature (e.g., Sostek, 1978; Munro et al., 1987; Vossel, 1988; Wilson and Graham, 1989; Michael et al., 2012).

To assess whether TSD influenced affect, sleepiness, vigilant attention, and EDA outcomes, we ran separate mixed-effects ANCOVAs on PANAS positive and negative affect, KSS sleepiness ratings, PVT LSNR, NSSCR frequency, and SCL. All models included fixed effects of condition (WRC, TSD), day (baseline, intervention day), time of day (morning, afternoon), and their interactions, and a random effect over participants on the intercept; protocol (1, 2, or 3) and participant sex (male, female) were included as covariates. To explore whether lability classification moderated the effect of TSD on EDA, we conducted equivalent analyses on the frequency of NSSCR and SCL with additional fixed effects of lability classification (stable, labile) and its interactions with the condition, day, and time of day. Although EDA data are often not normally distributed (Society for Psychophysiological Research Ad Hoc Committee on Electrodermal Measures, 2012), the data presented here were not skewed enough to warrant transforming the data (absolute skew was less than 1 for NSSCR and less than 2 for SCL). Partial eta squared ($\eta_{p}^{2}$) values for effect size were calculated for significant effects. All pairwise comparisons in follow-up to significant interactions were Bonferroni-adjusted.

To determine if our dependent variables showed systematic interindividual differences across days including TSD, we performed a variance components analysis (Van Dongen et al., 2004b) and assessed the intraclass correlation coefficient (ICC) based on the mixed-effects ANCOVA model specified above (without lability classification as a covariate). The ICC, calculated as the ratio of the between-subjects variance to the between- and within-subjects variance, quantified the extent to which variability in the observations was explained by systematic interindividual differences. ICC values range from 0 to 1, representing the stability of the interindividual differences from no systematic interindividual differences to perfectly stable interindividual differences. Standard errors and 95% confidence intervals were calculated using bootstrapping (1,000 simulations). To put the magnitude of interindividual differences in perspective, we calculated the absolute value of the ratio of the between-subjects standard deviation (square root of between-subjects variance) to the mean change from the baseline to the intervention day in the TSD condition.

To examine if a change in NSSCR frequency or SCL was related to the effect of TSD on affect, sleepiness, or vigilant attention, we calculated change scores from the baseline day to the TSD intervention day for all variables of interest, for the morning and afternoon times separately. We then ran separate mixed-effects ANCOVAs on the change scores for positive affect, negative affect, sleepiness, and LSNR as a dependent variable, using only data from the TSD participants. The models had a fixed effect for time of day (morning, afternoon) with a random effect over participants on the intercept. Change in EDA measure, protocol (1, 2, 3), and participant sex (male, female) were included as covariates. To determine whether a change in EDA was significantly related to the TSD effect, each model was compared to a reduced model without the change in EDA covariate using the variance ratio test, and the partial correlation (*r*_partial_) between EDA and the dependent variable was assessed. To supplement our findings, we conducted follow-up variance ratio tests using similar mixed-effects ANCOVA models using data from the baseline day only in both the WRC and TSD conditions to determine whether EDA accounted for significant variance in our dependent variables at baseline.

## Results

Descriptive statistics for the dependent variables are presented by condition, day, and time of day in Table 1.

**Table 1:** Descriptive statistics for all dependent variables by condition, day, and time of day.

Condition	Dependent variable	Time of day	Day 2			Day 3		
			Mean	SD	Range	Mean	SD	Range
WRC	Positive affect	Morning	26.62	7.62	11.00–47.00	24.29	8.11	10.00–47.00
		Afternoon	24.16	7.81	11.00–45.00	23.02	8.12	10.00–43.00
							
	Negative affect	Morning	11.05	2.01	10.00–22.00	10.86	1.60	10.00–17.00
		Afternoon	11.20	2.03	10.00–21.00	10.98	1.64	10.00–19.00
							
	Sleepiness	Morning	3.06	1.43	0.00–6.00	2.94	1.30	0.00–8.00
		Afternoon	3.08	1.03	1.00–6.00	2.97	0.73	1.00–5.00
							
	LSNR	Morning	14.46	1.53	11.15–17.92	13.92	1.59	9.95–19.47
		Afternoon	13.76	1.52	10.47–17.18	13.82	1.41	10.73–16.36
							
	Frequency of NSSCR	Morning	0.31	0.10	0.08–0.50	0.30	0.11	0.07–0.47
		Afternoon	0.28	0.11	0.02–0.48	0.28	0.12	0.03–0.53
							
	SCL	Morning	3.94	1.86	0.79–8.89	3.63	1.94	1.05–8.61
		Afternoon	3.50	2.06	0.53–10.14	3.35	1.81	0.81–10.68
								
TSD	Positive affect	Morning	27.47	8.65	11.00–50.00	16.71	6.79	10.007–43.00
		Afternoon	26.21	8.39	10.00–50.00	19.88	7.89	10.00–44.00
							
	Negative affect	Morning	11.10	1.86	10.00–21.00	11.99	2.90	10.00–26.00
		Afternoon	11.08	1.65	10.00–18.00	11.62	2.40	10.00–24.00
							
	Sleepiness	Morning	2.94	1.39	0.00–7.00	6.08	1.88	0.00–9.00
		Afternoon	2.72	1.11	0.00–6.00	5.46	1.97	0.00–9.00
							
	LSNR	Morning	14.55	1.38	11.47–19.75	11.38	2.37	5.31–17.83
		Afternoon	13.76	1.30	10.94–16.83	11.87	2.14	6.30–17.57
							
	Frequency of NSSCR	Morning	0.28	0.12	0.00–0.48	0.22	0.13	0.00–0.45
		Afternoon	0.24	0.11	0.00–0.50	0.22	0.12	0.00–0.47
							
	SCL	Morning	4.02	2.56	0.52–15.52	3.41	2.26	0.39–12.70
		Afternoon	3.51	2.04	0.34–9.81	3.26	1.94	0.29–8.92

### Effects of Total Sleep Deprivation

**Figure 1:**
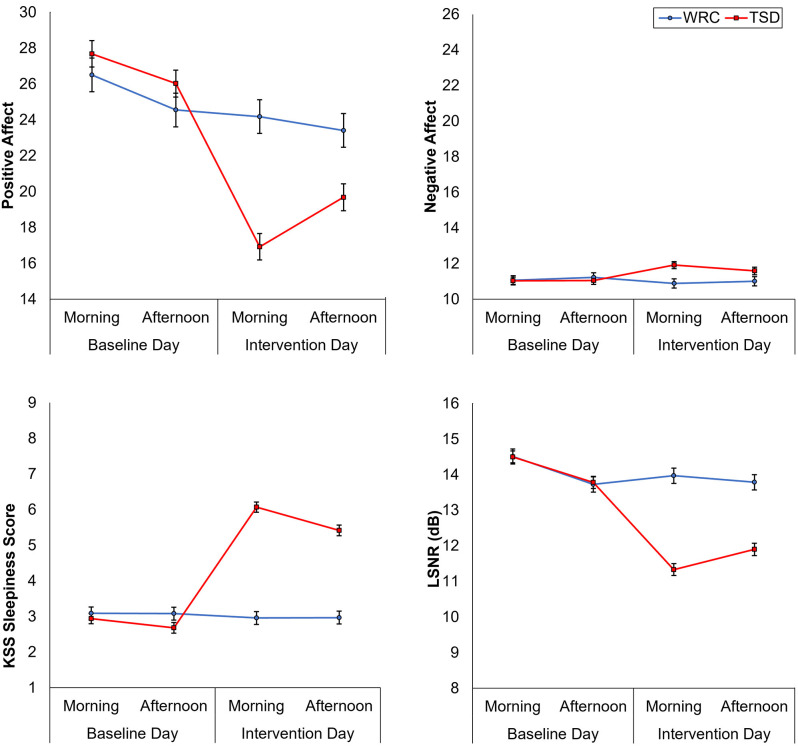
Changes in positive and negative affect (top row), subjective sleepiness, and vigilant attention performance (bottom row), from the baseline day (morning and afternoon) to the intervention day (morning and afternoon). Error bars represent ±1 standard error of the mean.

Figure 1 shows the effect of TSD on positive and negative affect, as well as sleepiness and PVT performance. Table 2 displays the results of the corresponding statistical analyses, where the condition by day interaction is of particular interest. In agreement with earlier findings (Franzen et al., 2008; Talbot et al., 2010; Riedy et al., 2013), TSD decreased positive affect and increased negative affect (*p* < 0.001), although the impact on negative affect was very small. Also, as expected, TSD increased subjective sleepiness and degraded vigilant attention performance on the PVT (*p* < 0.001). The impact of TSD was somewhat greater in the morning of the intervention day than in the afternoon, in accordance with the known effect of circadian rhythmicity during TSD (Skeiky et al., 2021). Sex was a significant covariate for negative affect, with males exhibiting greater negative affect than females. The stability of interindividual differences in affect, sleepiness, and vigilant attention performance ranged from 0.34 to 0.75, as shown in Table 3. The interindividual differences were smaller than the magnitude of the group-mean TSD effect for positive affect, sleepiness, and vigilant attention, and nearly twice as large as the group-mean TSD effect for negative affect. For negative affect, therefore, systematic interindividual differences were the dominant source of variability in this data set.

**Table 2:** Mixed-effects ANOVAs for positive and negative affect, subjective sleepiness, and PVT LSNR.

Dependent variable	Effect	*F*	df	*p*	$\eta_{p}^{2}$
Positive affect	Condition	3.87	1,467	<0.050	
	**Day**	**267.47**	**1,467**	**<0.001**	**0.36**
	Time of day	1.46	1,467	<0.228	
	**Condition × Day**	**117.62**	**1,467**	**<0.001**	**0.20**
	**Condition × Time of day**	**8.01**	**1,467**	<**0.005**	**0.02**
	**Day × Time of day**	**19.90**	**1,467**	**<0.001**	**0.04**
	**Condition × Day × Time of day**	**6.66**	**1,467**	<**0.010**	**0.01**
	Protocol	0.09	2,467	<0.917	
	Sex	2.70	1,467	<0.101	
					
Negative affect	Condition	2.14	1,467	<0.144	
	**Day**	**4.02**	**1,467**	<**0.046**	**0.01**
	Time of day	0.01	1,467	<0.943	
	**Condition × Day**	**12.85**	**1,467**	**<0.001**	**0.03**
	Condition × Time of day	1.27	1,467	<0.261	
	Day × Time of day	0.55	1,467	<0.457	
	Condition × Day × Time of day	0.37	1,467	<0.542	
	**Protocol**	**3.25**	**2,467**	<**0.040**	**0.01**
	**Sex**	**4.22**	**1,467**	<**0.041**	**0.01**
					
Sleepiness ratings	**Condition**	**60.40**	1,467	**<0.001**	**0.12**
	**Day**	**219.71**	1,467	**<0.001**	**0.32**
	**Time of day**	**5.16**	**1,467**	<**0.024**	**0.01**
	**Condition × Day**	**257.47**	1, 467	**<0.001**	**0.36**
	**Condition × Time of day**	**5.33**	**1,467**	<**0.021**	**0.01**
	Day × Time of day	1.02	1,467	<0.313	
	Condition × Day × Time of day	1.17	1,467	<0.280	
	Protocol	0.07	2,467	<0.933	
	Sex	2.39	1,467	<0.123	
					
LSNR	**Condition**	**30.69**	**1,467**	**<0.001**	**0.06**
	**Day**	**180.07**	**1,467**	**<0.001**	**0.28**
	**Time of day**	**6.63**	**1,467**	<**0.010**	**0.01**
	**Condition × Day**	**122.93**	**1,467**	**<0.001**	**0.21**
	Condition × Time of day	3.57	1,467	<0.060	
	**Day × Time of day**	**20.98**	**1,467**	**<0.001**	**0.04**
	Condition × Day × Time of day	2.74	1,467	<0.098	
	Protocol	0.45	2,467	<0.636	
	Sex	0.44	1,467	<0.509	

*Note. Bold indicates *p* < 0.05*.

**Table 3:** Intraclass correlation coefficient analyses.

	VAR_bs_ (SE)	VAR_ws_ (SE)	ICC [95% CI]	Between-subjects SD as a Proportion of the TSD effect
Positive affect	45.89 (5.35)	15.61 (1.00)	0.75 [0.69, 0.79]	0.79
Negative affect	1.77 (0.27)	2.58 (0.17)	0.41 [0.33, 0.49]	1.86
Sleepiness ratings	0.73 (0.12)	1.43 (0.09)	0.34 [0.25, 0.43]	0.29
LSNR	1.33 (0.20)	1.68 (0.11)	0.44 [0.37, 0.53]	0.46
Frequency of NSSCR	0.008 (0.001)	0.006 (0.0004)	0.57 [0.49, 0.64]	2.23

*Note. VAR_bs_, between-subjects variance; VAR_ws_, within-subjects variance; ICC, intraclass correlation coefficient; SE, standard error; CI, confidence interval*.

**Figure 2:**
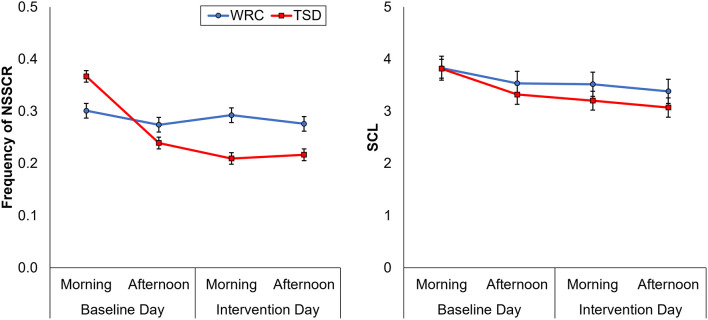
Changes in frequency of non-specific skin conductance responses (NSSCR; left) and skin conductance level (SCL; right), from the baseline day (morning and afternoon) to the intervention day (morning and afternoon). In each panel, downward corresponds to less physiological arousal; error bars represent ±1 standard error of the mean.

Figure 2 shows the effects of TSD on NSSCR frequency and SCL, and Table 4 displays the results of the corresponding statistics. The condition by day interaction is again of particular interest. In line with previous work (Posada-Quintero et al., 2017, 2018), TSD decreased the frequency of NSSCR (*p* = 0.003), although the effect was small. Unexpectedly, TSD did not have a significant effect on SCL (*p* = 0.273). Both the frequency of NSSCR and SCL were influenced by time of day, such that participants’ frequency of NSSCR and SCL were higher in the morning than in the afternoon. Retaining only the frequency of NSSCR for further analyses, we evaluated whether lability moderated the effect of TSD on this measure of EDA. As shown in Table 5, adding lability classification to our analysis did not change the pattern of results, nor did lability significantly moderate the TSD effect. There were stable, systematic interindividual differences in the frequency of NSSCR; see Table 3. The magnitude of these interindividual differences was considerably larger than the group-mean TSD effect, indicating that interindividual differences were the dominant source of variability in our NSSCR data.

**Table 4:** Mixed-effects ANOVAs for frequency of NSSCR and SCL.

Dependent variable	Effect	*F*	df	*p*	$\eta_{p}^{2}$
NSSCR	**Condition**	**13.54**	**1,467**	**<0.001**	**0.03**
	**Day**	**12.93**	**1,467**	**<0.001**	**0.03**
	**Time of day**	**6.32**	**1,467**	<**0.012**	**0.01**
	**Condition × Day**	**9.27**	**1,467**	<**0.003**	**0.02**
	Condition × Time of day	0.80	1,467	<0.371	
	Day × Time of day	3.51	1,467	<0.062	
	Condition × Day × Time of day	1.04	1,467	<0.308	
	**Protocol**	**4.87**	**2,467**	<**0.008**	**0.02**
	Sex	1.38	1,467	<0.241	
					
SCL	Condition	0.74	1,467	<0.389	
	**Day**	**13.11**	**1,467**	**<0.001**	**0.03**
	**Time of day**	**7.32**	**1,467**	<**0.007**	**0.02**
	Condition × Day	1.21	1,467	<0.273	
	Condition × Time of day	0.27	1,467	<0.605	
	Day × Time of day	2.03	1,467	<0.155	
	Condition × Day × Time of day	0.33	1,467	<0.568	
	**Protocol**	**26.08**	**2,467**	**<0.001**	**0.10**
	Sex	2.91	1,467	<0.089	

*Note. Bold indicates *p* < 0.05*.

**Table 5:** Mixed-effects ANOVA for frequency of NSSCR with lability classification.

Effect	*F*	df	*p*	$\eta_{p}^{2}$
**Condition**	**13.24**	**1,459**	**<0.001**	**0.03**
**Day**	**13.60**	**1,459**	**<0.001**	**0.03**
**Time of Day**	**6.23**	**1,459**	<**0.013**	**0.01**
**Condition × Day**	**13.69**	**1,459**	**<0.001**	**0.03**
Condition × Time of day	0.12	1,459	<0.725	
Day × Time of day	3.59	1,459	<0.059	
Condition × Day × Time of day	1.20	1,459	<0.274	
**Lability**	**265.53**	**1,459**	**<0.001**	**0.37**
Lability × condition	<0.01	1,459	<0.945	
**Lability × Day**	**17.45**	**1,459**	**<0.001**	**0.04**
Lability × Time of day	2.18	1,459	<0.140	
Lability × Condition × Day	0.27	1,459	<0.603	
Lability × Condition × Time of day	1.29	1,459	<0.256	
Lability × Day × Time of day	0.11	1,459	<0.737	
Lability × Condition × Day × Time of day	<0.01	1,459	<0.999	
**Protocol**	**6.16**	**2,459**	<**0.002**	**0.03**
Sex	0.04	1,459	<0.849	

*Note. Bold indicates *p* < 0.05*.

### NSSCR as a Predictor of TSD Effects

**Figure 3:**
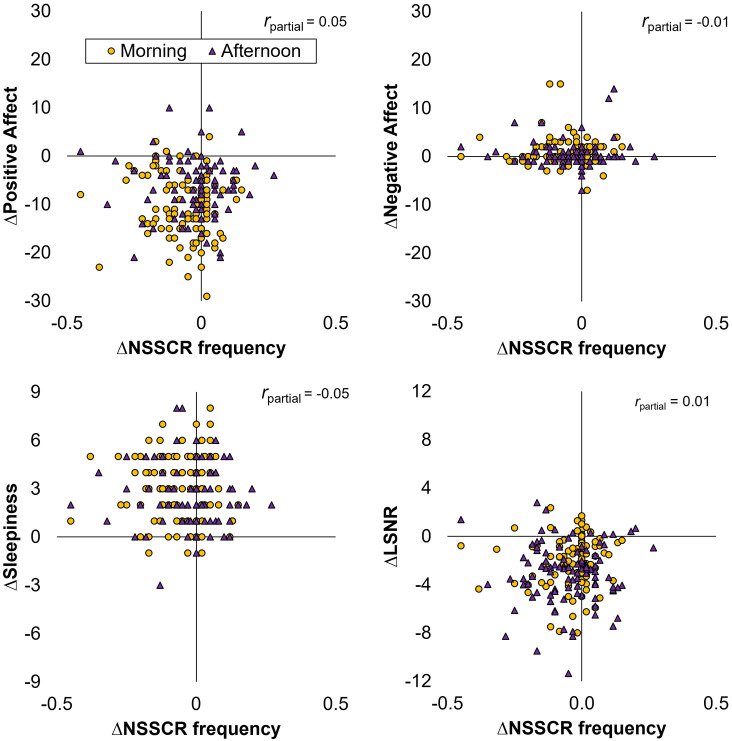
Scatter plots of the change across days (∆) in the frequency of NSSCR vs. positive affect (top left), negative affect (top right), subjective sleepiness (bottom left), and LSNR (bottom right) for participants in the TSD condition.

Focusing attention on the TSD condition only, we analyzed the change from the baseline day to the intervention day in each of the outcome variables of interest (affect, sleepiness, vigilant attention, and NSSCR) and investigated whether the change in frequency of NSSCR predicted the change in any of the other variables. As is evident from Figure 3, we found that the change in frequency of NSSCR was not a significant predictor for the change in any of the other outcome variables (positive affect: *F*_(1,83)_ = 1.27, *p* = 0.263; negative affect: *F*_(1,83)_ = 1.01, *p* = 0.317; subjective sleepiness: *F*_(1,83)_ = 1.20, *p* = 0.277; and LSNR:* F*_(1,83)_ = 1.01, *p* = 0.317). Thus, EDA as measured by the frequency of NSSCR, although by itself sensitive to TSD, did not predict the TSD-related change in self-reported affect, nor did it predict the change in our other measures reflecting arousal, subjective sleepiness, and vigilant attention. This contrasted with relationships among the non-EDA variables; we found that TSD-induced change in positive affect was a significant predictor for sleepiness (*F*_(1,83)_ = 10.31, *p* = 0.002, *r*_partial_ = -0.31) and vigilant attention (*F*_(1,83)_ = 5.03, *p* = 0.028, *r*_partial_ = 0.19), such that decreases in positive affect were associated with increased sleepiness and decreased vigilant attention. These analyses were repeated after removing data points with zero NSSCR to confirm the robustness of the findings^1^.

To determine whether the lack of relationship between NSSCR and the other variables during TSD was foreshadowed by a lack of relationship at baseline, we examined whether frequency of NSSCR predicted WRC and TSD participants’ baseline affect, sleepiness, and vigilant attention. Contrary to expectation, we found that frequency of NSSCR was not a significant predictor at baseline on any of our outcome variables (positive affect: *F*_(1,138)_ = 0.82, *p* = 0.366; negative affect: *F*_(1,138)_ = 2.08, *p* = 0.152; subjective sleepiness: *F*_(1,138) =_1.27, *p* = 0.261; and LSNR: *F*_(1,138)_ = 1.32, *p* = 0.253).

## Discussion

In our investigation of EDA and its relationship to changes in the emotional state during sleep loss, we found that TSD led to the expected decrease in positive affect, a small increase in negative affect, an increase in sleepiness, and the degradation of vigilant attention performance. Of the two EDA variables investigated, only the frequency of NSSCR (but not SCL) showed an effect of TSD, and this effect was not moderated by lability classification. Positive and negative affect, subjective sleepiness, and vigilant attention performance, as well as NSSCR, all displayed relatively stable, systematic individual differences, even during TSD. Importantly, though we observed the expected effects of TSD on affect, sleepiness, vigilant attention, and NSSCR, we found no evidence that TSD-induced changes in physiological arousal as measured by the frequency of NSSCR predicted the TSD effects on the other variables. Overall, these findings indicate that although EDA may be useful for assessing TSD-related changes in non-specific arousal at the group level, it does not appear to predict TSD-induced changes in self-reported affect, nor subjective sleepiness or vigilant attention, at the level of individuals. This suggests that there is an essential aspect of the relationship between physiological arousal and self-reported affect that is not well captured by EDA as measured by NSSCR.

Confidence in the findings of this study is bolstered by several factors. First, we used a large sample of participants. Compared to previous studies of sleep deprivation and EDA, which generally have around 10–30 participants undergo sleep deprivation, our sample had over 100 in the TSD condition alone. This large sample minimizes the chances that a single individual could have greatly influenced our results—a particularly important factor considering the significant interindividual differences we observed across all our measures. Second, we had extensive screening measures in place, and the data were collected under strict laboratory control. Participants in the laboratory were monitored during their entire stay and were not allowed to engage in behaviors that may distort the findings (e.g., making phone calls or drinking caffeine). Third, unlike most recent studies of EDA and sleep deprivation (cf. Liu et al., 2015), this study also included a well-rested control, which allowed us to account for how time spent in the laboratory alone affected our measures. Fourth, using data from the same morning and afternoon time points on both days allowed us to account for circadian rhythm in our comparisons. Finally, unlike most previous studies of EDA and TSD, we accounted for interindividual differences, which we found to be systematic and substantial in nature. This allowed us to go beyond the group-level effects of TSD in our analyses and revealed the dissociation between EDA and other measures at the level of individuals.

Despite these strengths, our findings are limited by the fact that we included only data from two time points per day. Other studies of EDA during sleep deprivation generally collect measures of interest, such as EDA, affect, sleepiness, or PVT performance, across the circadian cycle (see Miró et al., 2002; Michael et al., 2012; Posada-Quintero et al., 2018), as do studies of interindividual differences (e.g., Van Dongen et al., 2004a, b; Lundholm et al., 2021). Because this study retrospectively analyzed previously collected data, we were only able to gather SCL and NSSCR data during time periods immediately preceding when SCR recording had taken place, which were limited to periods intended to study task-specific performance at two designated time intervals during the daytime hours. In a related vein, our use of retrospective data constrained what measures we could use to investigate affect, as only PANAS data were available across all three protocols. Although the PANAS is commonly used in sleep deprivation studies (Franzen et al., 2008; Riedy et al., 2013; Stenson et al., 2021), it does not allow affect to be decomposed by both arousal and valence, which may have limited our ability to detect a relationship between EDA and affect during TSD. Finally, although the three protocols we used here had similar procedures, they were run in different years. Changes to procedures, staff, and the demographic makeup of the local population may have all contributed to differences we observed between protocols and, although controlled for in statistical analyses, may have added noise to our data.

Despite the limitations, we replicated the well-reported group-level effects of TSD on positive and negative affect, on subjective sleepiness, and on vigilant attention performance. Further, we replicated the group-level effect of TSD on the frequency of NSSCR, such that the frequency of NSSCR diminished when individuals were sleep deprived (Michael et al., 2012; Posada-Quintero et al., 2017, 2018). At the individual level, we replicated the finding that individuals vary in their vulnerability to sleep deprivation. Positive and negative affect, subjective sleepiness, and vigilant attention performance exhibited systematic interindividual differences under sleep deprivation. The stability of these differences ranged from rather low (for sleepiness ratings) to moderate (for negative affect and LSNR) to rather high (for positive affect), with ICC values that were lower than found previously when nighttime measurements were also included in analyses (Van Dongen et al., 2004a). Furthermore, we expanded upon previous work reporting interindividual differences in EDA (Crider, 1993; Crider et al., 2004). Although previous investigations of the stability of the frequency of NSSCR were done using twin studies or using healthy rested adults (Isen et al., 2012; Vaidyanathan et al., 2014; Bari, 2019), to our knowledge this is the first study to document interindividual differences in the frequency of NSSCR under sleep deprivation. We found that these interindividual differences are moderately stable and considerably greater than the group-mean effect of one night of TSD on NSSCR.

In contrast to previous work, at the group level, we did not find a significant effect of TSD on SCL. In one study the skin resistance level, which is the inverse of SCL, was found to increase with time spent awake (Miró et al., 2002), but other work showed that SCL is less sensitive to sleep loss than NSSCR, a higher frequency measure of EDA (Posada-Quintero et al., 2017, 2018). Studies of TSD and EDA that observed a TSD effect on SCL also collected data during nighttime wakefulness, when the effect of TSD is amplified by circadian rhythm. We may have failed to observe a significant effect of TSD on SCL because we considered only the daytime measurements shared between the TSD and rested control conditions. Separately, we did not find that participants’ baseline lability classification significantly moderated the effect of TSD on the frequency of NSSCR, as the interaction of lability, condition, and day was not significant. However, lability did moderate the effect of day in the study, such that regardless of whether participants were sleep-deprived, stable participants’ frequency of NSSCR did not significantly change across days, whereas labile participants had fewer NSSCR on the intervention day than at baseline. As the study that originally reported a moderating influence of lability on the effect of TSD on NSSCR (Michael et al., 2012) did not have a rested control condition, it is possible that the investigators misattributed a non-specific effect of day on NSSCR to increasing time awake. Alternatively, the lack of a moderating effect of lability classification on TSD may reflect differences in the dose of sleep deprivation in our study, which measured EDA until around 30 h awake, vs. the original work that did find a moderation effect, which measured EDA until around 51 h awake (Michael et al., 2012).

Critically, across individuals, the change in frequency of NSSCR was not significantly related to TSD participants’ self-reported affect. We expected EDA to be associated with participants’ self-reported affect, as SCL and frequency of NSSCR have been linked to emotion regulation (Gross and Levenson, 1993; Gross, 1998; Egloff et al., 2006; Duijndam et al., 2020) and to ratings of arousal in response to emotional stimuli (Gomez et al., 2016; Rattel et al., 2020; Sato et al., 2020). Yet all of these studies included an emotional induction through picture or film stimuli, whereas we measured changes in affect over time spent awake. While TSD did influence participants’ affect, it is not necessarily a reliable form of emotion induction. As emotions consist of shorter-lived, specific experiences elicited from a given stimulus, our lack of a relationship at rested baseline between frequency of NSSCR and affect may reflect a difference between measuring affect and measuring emotion (Russell, 2009). It would be of interest to investigate whether TSD would alter EDA and experienced emotion responses to emotional induction stimuli (cf. Franzen et al., 2008; Stenson et al., 2021).

Studies that observed a relationship between the frequency of NSSCR and experienced emotion under rested conditions (Gomez et al., 2016; Rattel et al., 2020; Sato et al., 2020) generally found a relationship with the arousal but not the valence dimension of emotion. Our use of the PANAS, which has arousal and valence intertwined, may not have been optimal to detect the relationship between the frequency of NSSCR and the arousal dimension of emotion. The much smaller effect of TSD on negative affect as compared to positive affect may have also contributed to that issue. However, we also did not find a change in frequency of NSSCR to be significantly related to the TSD effects on self-reported sleepiness and vigilant attention performance, our well-established subjective and objective correlates of the reduction in physiological arousal during sleep deprivation (Doran et al., 2001; Åkerstedt et al., 2014). This is in contrast to significant correlations obtained in previous studies of EDA and sleep deprivation (Miró et al., 2002; Posada-Quintero et al., 2018), which collected EDA measures during (rather than before or after) the performance of a vigilance task (Miró et al., 2002; Posada-Quintero et al., 2018). This likely increased physiological arousal and may have exposed a possible relationship between EDA and vigilant attention performance during sleep deprivation that remained concealed in our investigation based on a more passive measurement of EDA. Our findings are also at odds with research that has repeatedly found differences in vigilant attention performance by lability classification (Crider and Adgenbraun, 1975; Sostek, 1978; Munro et al., 1987). Although these studies measured EDA before engaging in a vigilance task, they focused on group-level analyses. It is unclear whether these same effects would be observed at the individual level.

This study represents an advancement in the understanding of how TSD effects on physiological arousal relate to TSD effects on affective and cognitive outcomes. Our findings are largely consistent with previous work when examining group differences, but we do not find any significant relationship between the frequency of NSSCR and self-reported affect, sleepiness, or vigilant attention at the level of individuals at baseline or during TSD. Our findings suggest that the well-documented TSD effects on physiological arousal on the one hand and affect, sleepiness, and vigilant attention, on the other hand, are not reflections of a single underlying phenomenon, despite being conceptually linked. While sleep deprivation has been shown to influence both sympathetic and parasympathetic arousal (McEwen, 2006), EDA reflects changes in sympathetic arousal specifically. Therefore, it is possible that TSD changes in parasympathetic arousal may be related to affect, which would explain the dissociation between affect and EDA.

At first glance, it seems problematic that the frequency of NSSCR did not account for significant variance in any of the measures that have been previously associated with EDA, but subjective and cognitive measures obtained under conditions of sleep deprivation often do not cluster with physiological measures across individuals (Leproult et al., 2003; Van Dongen et al., 2004a; Franzen et al., 2008). That we did not find a relationship during TSD between physiological arousal and affect in particular, and also no significant moderating effect of lability classification, does not support the proposition by some that lability is related to individuals’ ability to regulate their emotions (Crider, 2008). TSD has been found to reduce available cognitive resources at an individual’s disposal for given cognitive processes (Drummond et al., 2001; Chee and Van Dongen, 2013; Sullan et al., 2021). It would be reasonable, then, to assume that those individuals whose frequency of NSSCR is more strongly diminished by TSD may have fewer resources to regulate their mood. That should be reflected in their self-reported positive and negative affect, which is not what we found. However, it is important to note that this study was not specifically designed to assess the relationship between lability and emotion regulation.

Overall, NSSCR may be a useful tool for assessing sleep deprivation effects on non-specific arousal, particularly for those interested in monitoring arousal in operational settings and remote environments where decreases in alertness may have negative impacts on productivity or health and safety. However, this measure is unlikely to be predictive of TSD-related changes in an individual’s subjective emotional state or subjective sleepiness, or vigilant attention. Organizations interested in using physiological measures to detect negative emotions or other mental states in their employees (e.g., commanders monitoring warfighters, or flight surgeons monitoring astronauts) should be aware of the limitations of using EDA to this end. Further, our finding that physiological arousal as measured by EDA was not associated with multiple measures expected to be sensitive to arousal reduction during sleep deprivation suggests that measures of physiological arousal are dissociable from affective and cognitive measures during TSD. There is a need for more research into the role of sleep and sleep loss with regard to neurophysiological mechanisms underlying experienced emotion.

## Data Availability Statement

The raw data supporting the conclusions of this article will be made available by the authors, without undue reservation.

## Ethics Statement

The studies involving human participants were reviewed and approved by Washington State University Institutional Review Board. The participants provided their written informed consent to participate in this study.

## Author Contributions

JH, CK, PW, AS, and HVD contributed to the conception and design of the study. JH and CK organized the database, and CK performed the statistical analyses. CK and AS wrote the first draft of the manuscript. All authors contributed to the article and approved the submitted version.

## Conflict of Interest

The authors declare that the research was conducted in the absence of any commercial or financial relationships that could be construed as a potential conflict of interest.

## Publisher’s Note

All claims expressed in this article are solely those of the authors and do not necessarily represent those of their affiliated organizations, or those of the publisher, the editors and the reviewers. Any product that may be evaluated in this article, or claim that may be made by its manufacturer, is not guaranteed or endorsed by the publisher.

## Footnotes

^1^Removing the zero NSSCR cases from analyses did not fundamentally change the results found, with the effect of TSD on NSSCR still being present, *F*_(1,454)_ = 6.98, *p* = 0.009, and still no effect of TSD on SCL, *F*_(1,454)_ = 0.93, *p* = 0.334. Further, the subsequent analyses of how change in NSSCR influenced TSD effects on affect, sleepiness, and vigilant attention also showed the same pattern: positive affect, *F*_(1,77)_ = 1.07, *p* = 0.304; negative affect, *F*_(1,77)_ = 1.00, *p* = 0.321; subjective sleepiness *F*_(1,77)_ = 1.35, *p* = 0.249; and LSNR, *F*_(1,77)_ = 1.11, *p* = 0.295.
